# Thoracoacromial Pedicle-Supercharged Conjoined Flap With Cutaneous Advancement Flap and Pectoralis Major Integration for Chest Wall Reconstruction Following Multiple Recurrent Dermatofibrosarcoma Protuberans: A Case Report

**DOI:** 10.7759/cureus.89315

**Published:** 2025-08-04

**Authors:** Ioannis Kyriazidis, Athanasios Papas, Andreas Antoniades, Dimitrios Raptis, Leonidas Pavlidis

**Affiliations:** 1 Department of Plastic and Reconstructive Surgery, General Hospital Papageorgiou, Thessaloniki, GRC; 2 Faculty of Health Sciences, School of Medicine, Aristotle University of Thessaloniki, Thessaloniki, GRC; 3 Department of Plastic Surgery, School of Medicine, Aristotle University of Thessaloniki, Thessaloniki, GRC; 4 2nd Department of Surgery, Aristotle University of Thessaloniki, G. Gennimatas General Hospital, Thessaloniki, GRC

**Keywords:** advancement flap, chest wall reconstruction, dermatofibrosarcoma protuberans, dfsp, elderly patients, myocutaneous flap, pectoralis major flap, plastic surgery, recurrent sarcoma, thoracoacromial artery

## Abstract

Recurrent dermatofibrosarcoma protuberans (DFSP) of the anterior chest wall in elderly patients presents a complex reconstructive challenge, necessitating approaches that balance oncological radicality with minimized morbidity and optimal tissue quality for potential adjuvant radiotherapy. We report the case of an 84-year-old woman with an eighth recurrence of chest wall DFSP. Following wide local excision with clear margins, a significant soft tissue defect remained. To address the patient's advanced age, comorbidities, and need for robust tissue in a planned radiation field, an unconventional reconstructive approach was employed. A laterally based pectoralis major myocutaneous advancement flap was designed, incorporating the lateral remnant of the pectoralis major muscle and overlying skin. Crucially, the pectoral branch of the thoracoacromial artery pedicle was meticulously preserved and utilized to augment the flap's vascularity. This allowed for the strategic transfer of well-perfused muscle tissue into the defect, particularly to the area designated for postoperative radiation. The patient experienced an uneventful postoperative course with excellent flap viability and minimal donor site morbidity. The total operative time was 105 minutes. Adjuvant radiation therapy was well-tolerated by the robust myocutaneous flap. At six-month follow-up, there was no evidence of tumor recurrence, with favorable functional and aesthetic outcomes. The thoracoacromial artery-enhanced pectoralis major myocutaneous advancement flap offers a safe, efficient, and reliable reconstructive solution for complex chest wall defects in high-risk elderly patients with recurrent sarcomas. This technique minimizes operative burden while providing durable, well-vascularized tissue capable of withstanding adjuvant radiotherapy, thereby optimizing both oncological and reconstructive outcomes.

## Introduction

Cutaneous sarcomas, such as dermatofibrosarcoma protuberans (DFSP), represent a heterogeneous group of rare malignancies with a notable propensity for local recurrence, particularly after multiple treatment failures. Wide local excision with clear margins remains the cornerstone of curative-intent therapy for DFSP, often resulting in substantial soft tissue defects that necessitate complex reconstruction [1]. The reconstructive goals in such cases must meticulously balance oncological safety with functional and aesthetic outcomes while minimizing donor site morbidity and operative time. These considerations become paramount in elderly patients or those with significant comorbidities, where extensive procedures such as free tissue transfer or large pedicled flaps may carry prohibitive risks or increased morbidity [2,3].

The traditional reconstructive ladder, which advocates for the simplest solution first, has evolved into the "reconstructive elevator" concept, emphasizing that the optimal solution may not always be the least complex, especially when considering long-term durability, function, and aesthetic outcomes in challenging scenarios [4]. This philosophy has further progressed to embrace the "reconstructive toolbox" approach, where surgeons combine multiple reconstructive elements and techniques to create individualized solutions rather than selecting a single predetermined option [5]. Such innovative, personalized approaches often represent the optimal strategy for achieving durable coverage with minimal perioperative morbidity in challenging patient populations.

We present a case of an elderly patient with chest wall DFSP with multiple recurrences managed with wide excision and reconstruction using an unconventional technique: a laterally based cutaneous advancement flap incorporating part of the pectoralis major muscle augmented by the thoracoacromial pedicle. This approach successfully addressed the complex interplay of oncological requirements, patient-specific risks, and the need for robust tissue in a potentially irradiated field.

## Case presentation

**Figure 1:**
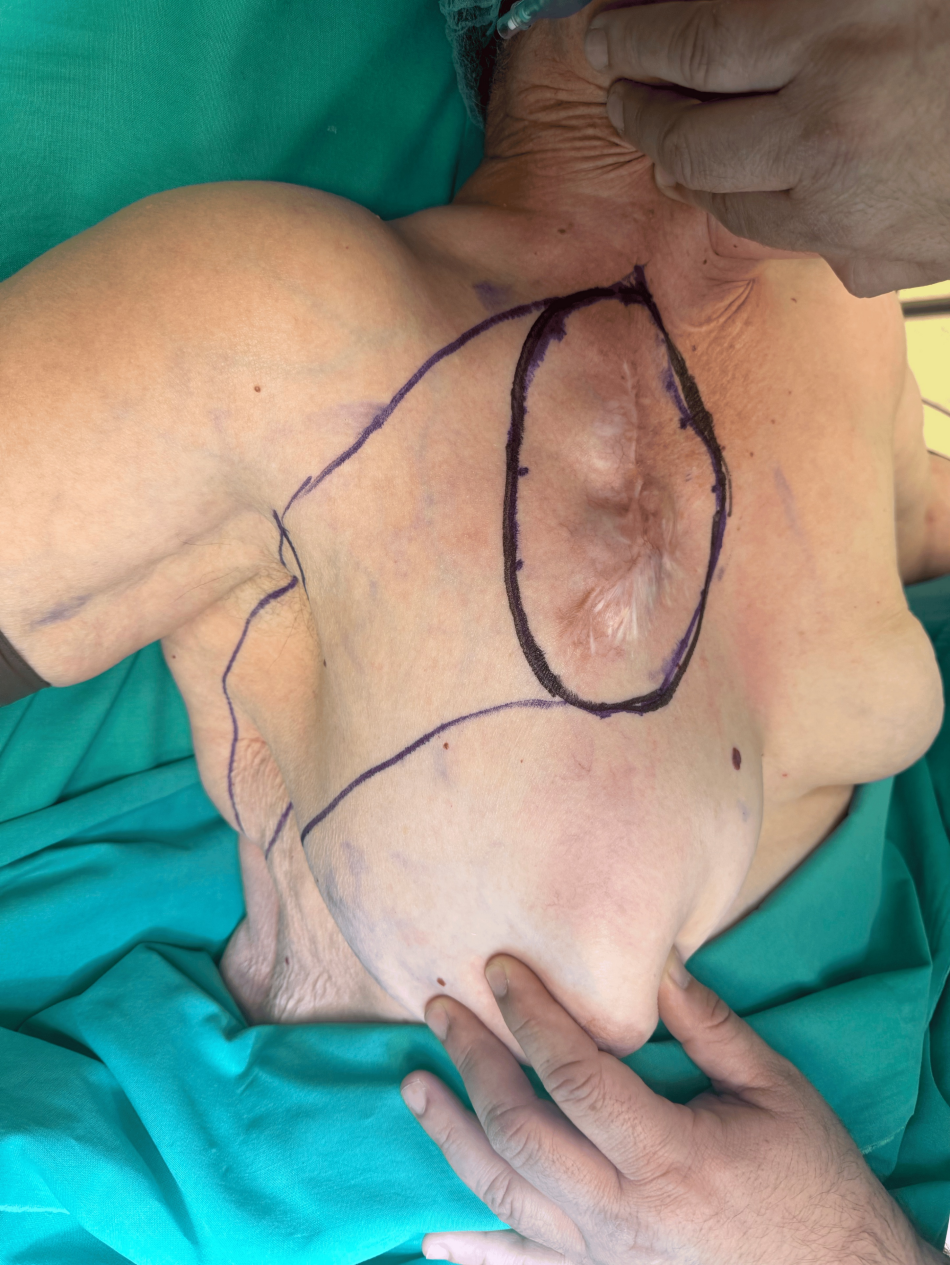
Preoperative markings showing the tumor with planned 3 cm margins.

An 84-year-old woman presented to our service on April 29, 2024, with her eighth recurrence of a dermatofibrosarcoma protuberans in the right anterior chest wall (Figure 1). The patient's disease history spanned nearly four decades, with initial diagnosis in 1986, followed by recurrence in 2008 (22 years later) and subsequent recurrence in 2020 (12 years later). From 2020 to early 2024, she experienced six additional recurrences at accelerating 4-6-month intervals, demonstrating an increasingly aggressive recurrence pattern. Her past medical history included solely hypertension, and she had undergone seven prior excisions of the sarcoma at the same site, with pathology consistently confirming recurrent DFSP. Preoperative staging with computed tomography (CT) confirmed the localized nature of the recurrence, with no evidence of distant metastatic disease.

**Figure 2:**
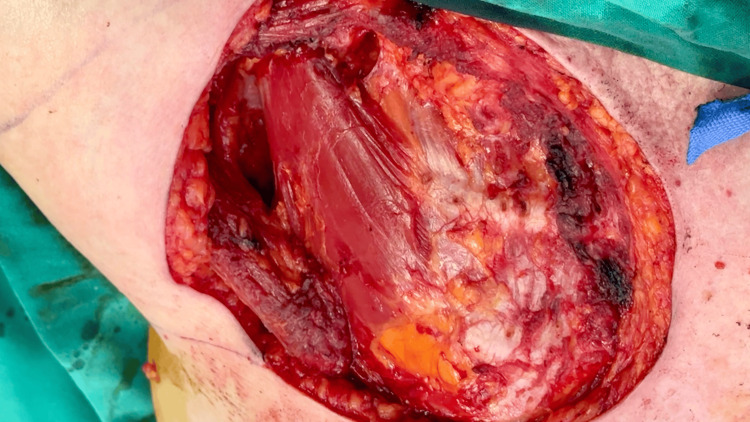
Intraoperative image after tumor excision showing the defect extending to the chest wall.

A multidisciplinary approach was essential in this complex case, with the team prioritizing minimal morbidity while ensuring adequate oncological margins and radiation-ready reconstruction. Surgical excision involved the resection of the recurrent lesion with 3 cm peripheral margins, extending down to the chest wall, including the underlying pectoralis major muscle beneath the peripheral margins (Figure 2). Intraoperative frozen section analysis confirmed clear, deep, and peripheral margins.

The resulting defect, measuring approximately 16 × 12 × 2 cm, presented a reconstructive challenge due to its size, the patient's age, and the need for a reliable, well-vascularized tissue in a potentially irradiated field. Reconstructive options were carefully considered, with the decision made to avoid more demanding procedures such as a latissimus dorsi (LD) myocutaneous flap.

Surgical technique

**Figure 3:**
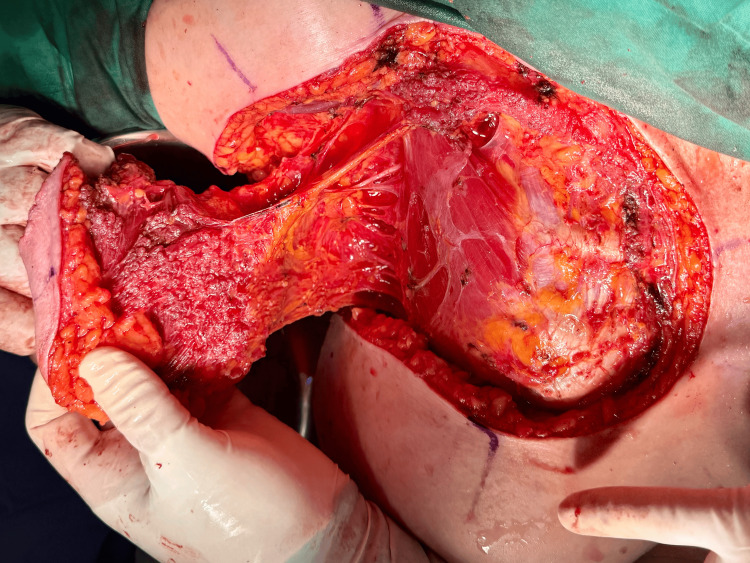
Identification and preservation of the thoracoacromial artery pedicle and its pectoral branch during flap elevation, inserting into the lateral aspect of the pectoralis major.

Following the confirmation of clear margins after wide local excision, the reconstructive phase commenced. A laterally based cutaneous advancement flap incorporating subcutaneous tissue and the remaining viable lateral portion of the pectoralis major muscle on its leading edge was designed. Careful dissection was performed to identify and preserve the thoracoacromial artery pedicle (Figure 3). This pedicle was meticulously preserved during flap elevation to augment the vascularity of the advancement flap.

The flap design incorporated the residual lateral segment of the pectoralis major muscle. This muscle component was included with the overlying skin and subcutaneous tissue, creating a myocutaneous flap. The insertion of the pectoralis major was divided at its musculotendinous junction, and the flap was then advanced medially to cover the central chest wall defect resulting from the tumor excision. The muscular portion of the flap was positioned into the deepest part of the defect and the area anticipated to receive radiotherapy.

**Figure 4:**
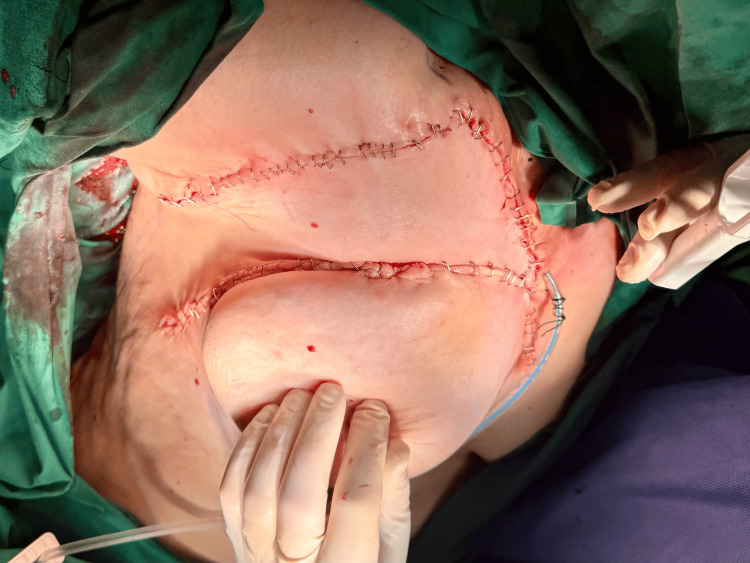
Final flap inset with primary donor site closure and drain placement.

The flap was inset without tension, and the donor site was closed primarily (Figure 4). Drains were placed as needed. The preservation of the thoracoacromial pedicle ensured robust perfusion to the advanced tissue, while the inclusion of the pectoralis muscle segment provided bulk and enhanced vascularity. The total operative time was 105 minutes, and the estimated blood loss was minimal at 50 mL.

Postoperative course

**Figure 5:**
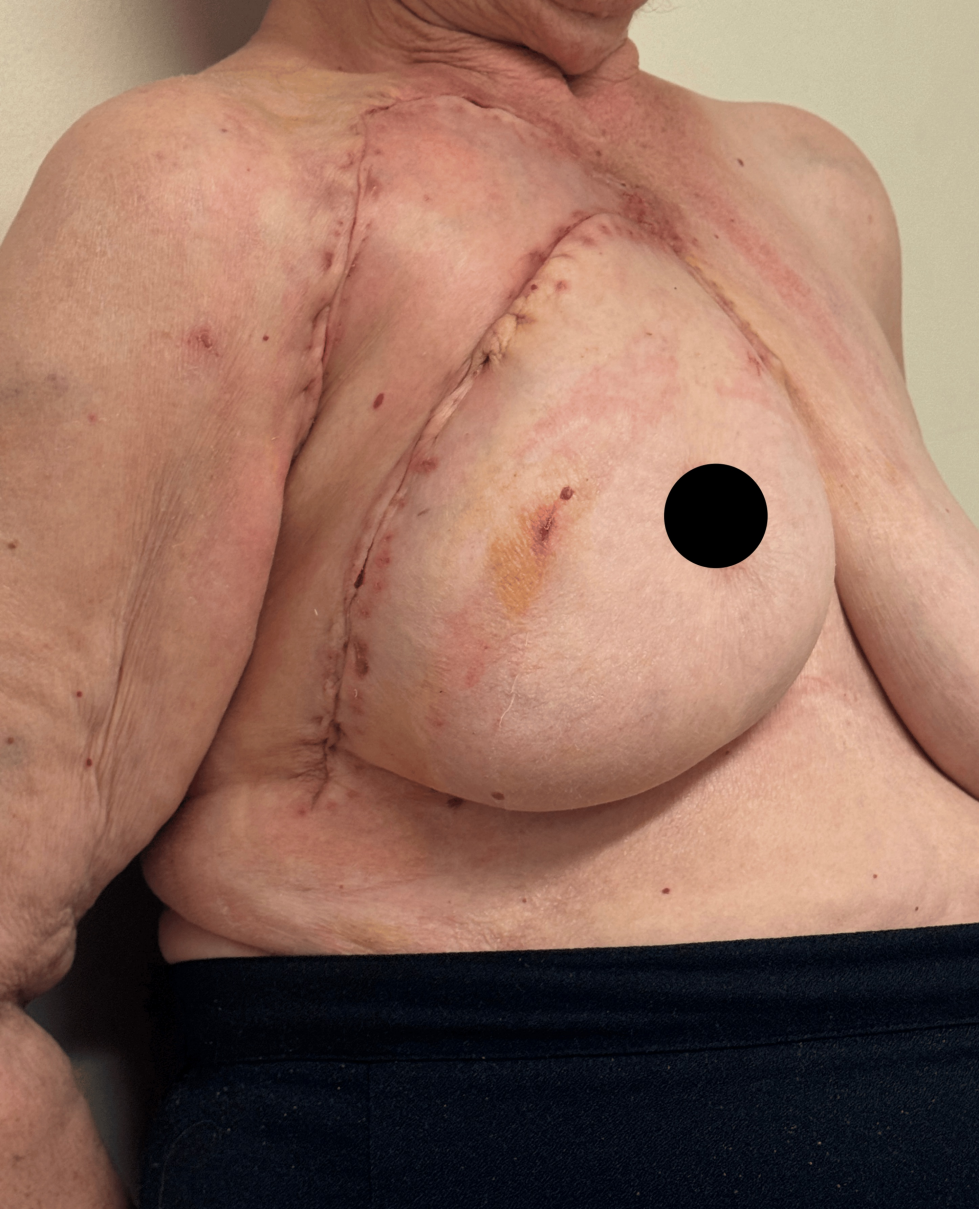
Three-week postoperative result showing fully healed incisions and no patient-reported issues during her everyday light activities.

The patient recovered uneventfully and was discharged on postoperative day 2. Follow-up at one week, two weeks, and one month showed progressive healing with no evidence of flap compromise or wound complications (apart from very small wound dehiscence on the most distal aspect of the flap to the thoracic area). The patient experienced minimal functional deficit and was able to resume most of her daily activities within three weeks of surgery, when the wound had already fully healed (Figure 5).

Adjuvant radiation therapy was initiated at six weeks postoperatively, delivering 60 Gy to the tumor bed. The myocutaneous nature of the flap provided robust coverage that withstood the radiation treatment without evidence of tissue breakdown or delayed wound healing complications.

**Figure 6:**
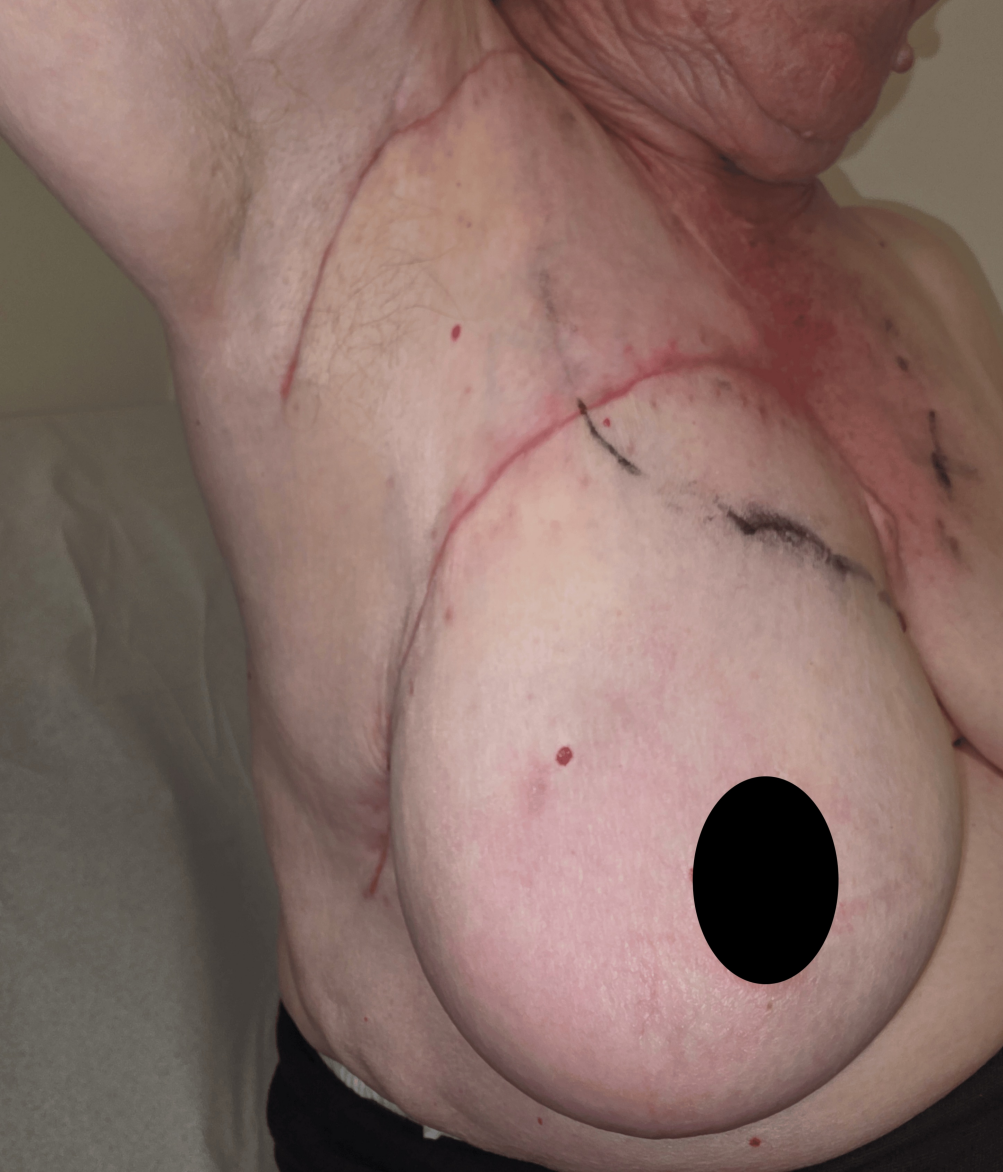
Six-month postoperative follow-up demonstrating the absence of tumor recurrence with excellent aesthetic and functional restoration. Minimal donor site morbidity was observed, and the patient reported high satisfaction with the surgical outcome.

At six-month follow-up, the patient showed no signs of tumor recurrence, with excellent aesthetic and functional outcomes (Figure 6). The donor site morbidity was minimal, and the patient reported high satisfaction with the procedure.

## Discussion

Managing multiple recurrent sarcomas, especially on the chest wall in elderly patients, presents a complex oncological and reconstructive dilemma. Achieving wide surgical margins is critical for local control, but the resultant defects often require robust reconstruction, particularly if adjuvant radiotherapy is planned. The 3 cm margins employed in this case align with current oncological guidelines recommending 2-4 cm margins for recurrent DFSP [6]. Radiotherapy should be considered when supplemental local control beyond surgical margins provides clinically relevant benefit, when patients face an increased likelihood of local recurrence, when the impact of recurrent disease would be substantial, or when the future management options for recurrence are constrained [7]. Such complex treatment decisions require multidisciplinary team collaboration to optimize both oncological and reconstructive outcomes. While free tissue transfer and large pedicled flaps such as the latissimus dorsi (LD) or rectus abdominis myocutaneous flaps offer reliable solutions, they are associated with significant operative time, potential donor site morbidity, and increased physiological demand, which may be poorly tolerated by elderly or frail individuals [8-10]. The decision to avoid the latissimus dorsi myocutaneous flap in this case was based on its associated donor site morbidity and extended operative time, particularly relevant considerations in elderly patients.

In this case, the patient's advanced age and history of seven prior recurrences necessitated a reconstructive strategy that prioritized safety, efficiency, and reliability. The primary goals were minimal patient morbidity, reduced anesthesia time, the preservation of more complex options such as the LD flap to reduce postoperative morbidity, and the provision of well-vascularized tissue capable of withstanding adjuvant radiotherapy. The chosen technique, an advancement cutaneous flap incorporating part of the pectoralis major augmented by the thoracoacromial artery pedicle, directly addressed these goals.

The use of a local advancement flap minimizes donor site morbidity compared to distant flaps [11]. Incorporating the residual lateral pectoralis major muscle provided several advantages over a simple fasciocutaneous advancement flap. The thoracoacromial pedicle, known for its robust blood supply to the pectoralis major muscle and overlying skin, was carefully preserved to "supercharge" or augment the vascularity of the advancement flap. This concept of enhancing an advancement flap with a known musculocutaneous perforator improves its safety and reliability.

Adjuvant radiation therapy was initiated at six weeks postoperatively, delivering 60 Gy to the tumor bed in 30 fractions over six weeks, which was consistent with established protocols for soft tissue sarcomas [12]. Furthermore, the strategic placement of the muscular component into the central defect, the anticipated field of radiotherapy, is significant. The successful tolerance of adjuvant radiation by the myocutaneous flap in this case is consistent with research regarding the superior radioresistance of the muscle versus the skin. Muscle tissue is generally more tolerant to radiation than skin and subcutaneous tissue alone, potentially reducing the risk of radiation-induced complications such as fibrosis or flap breakdown [13]. This approach involving a supercharged conjoined flap with a cutaneous advancement flap and pectoralis major integration, therefore, not only closed the defect but also optimized the tissue bed for subsequent adjuvant therapy.

It is important to acknowledge the limitations inherent in a single case report. While this technique demonstrated favorable outcomes in our patient, we do not claim superiority over established reconstructive methods such as free tissue transfer or traditional pedicled flaps. Larger case series with comparative analysis would be necessary to establish the relative efficacy, safety profile, and optimal patient selection criteria for this approach.

However, this case underscores the fundamental principle that reconstructive surgery, particularly in complex oncological scenarios, should be highly individualized rather than algorithmic. This case exemplifies the evolution from the traditional reconstructive ladder to the contemporary "reconstructive toolbox" concept, as described by Hallock [5]. Rather than selecting a single predetermined technique from the reconstructive hierarchy, our approach combined multiple reconstructive elements, a random pattern cutaneous advancement flap, thoracoacromial pedicle supercharging, and strategic pectoralis major integration, to create a bespoke solution tailored to this patient's specific requirements. This "toolbox" methodology allows surgeons to synthesize different reconstructive components, drawing from various techniques simultaneously rather than being constrained to a single option from the traditional ladder. The resulting conjoined flap with supercharging represents precisely this philosophy: utilizing whatever combination of tools is necessary to achieve optimal form and function for the individual patient's unique reconstructive challenge.

In elderly patients with recurrent disease, where the balance between oncological adequacy and physiological tolerance becomes critical, such tailored approaches may offer significant advantages in terms of morbidity, operative efficiency, and functional outcomes. The key lies in the surgeon's ability to innovatively adapt established principles to meet the unique demands of each clinical scenario while maintaining the technical expertise to execute more complex alternatives when indicated.

## Conclusions

The reconstruction of chest wall defects after the wide excision of recurrent sarcoma in elderly patients requires the careful consideration of morbidity, operative time, and tissue requirements for potential adjuvant therapy. We successfully utilized a pectoralis major mini-myocutaneous advancement flap, augmented by the thoracoacromial artery pedicle, to reconstruct a significant defect in an 84-year-old woman. This technique provided robust, well-vascularized coverage with minimal donor site morbidity and operative time while preserving more complex reconstructive options. This approach represents a valuable addition to the armamentarium for managing complex chest wall defects in selected high-risk patients.
